# Microbial Contamination and Molecular Identification of Predominant Bacteria in Fermented Milk Products in Mbarara, Uganda

**DOI:** 10.7759/cureus.108346

**Published:** 2026-05-06

**Authors:** Monicah Kamwine, Pauline Petra Nalumaga, Fredrickson B Wasswa, Karuhanga Naume Joyce, Immanuel Afrika Musau, Yap Boum II, Joel Bazira

**Affiliations:** 1 Microbiology and Parasitology, Mbarara University of Science and Technology, Mbarara, UGA; 2 Microbiology, Mbarara University of Science and Technology, Mbarara, UGA; 3 Public Health, Institut Pasteur de Bangui, Bangui, CAF

**Keywords:** dairy safety, fermented milk, foodborne pathogens, sanger sequencing, uganda

## Abstract

Background: Fermented milk products are widely consumed in Uganda for their nutritional and cultural value. However, informal production practices and limited quality control raise concerns regarding microbial safety. This study evaluated the microbiological safety of commonly consumed fermented dairy products in Mbarara City, Uganda.

Methods: A total of 192 samples, including yogurt, cheese, homemade butter, and *eshabwe* (a traditional butter sauce), were analysed. Culture-based testing was performed on 175 samples to detect *Escherichia coli, Staphylococcus aureus*, and *Salmonella spp*. Partial 16S rRNA gene sequencing (Sanger method) was conducted on 17 representative samples to identify predominant bacterial taxa.

Results: Overall, 12.0% of cultured samples were positive for at least one pathogen. Cheese had the highest contamination rate (42.9%), followed by eshabwe (8.6%) and yogurt (4.3%), while homemade butter showed no detectable pathogens. Molecular analysis confirmed the presence of fermentation-associated lactic acid bacteria and identified potential opportunistic and pathogenic taxa, including *Klebsiella spp* and *Enterobacter spp*.

Conclusions: Fermented dairy products in Mbarara City contain beneficial fermentative bacteria but also demonstrate measurable pathogen contamination, particularly in products under informal conditions. Targeted hygiene improvements, safe starter use, and routine microbial monitoring are needed to enhance food safety while preserving traditional production practices across Uganda

## Introduction

Milk and dairy products are integral to the diets of communities across sub-Saharan Africa. These products offer essential nutrients and contribute significantly to food security and livelihoods, particularly among pastoral populations [1,2]. Among these, fermented milk products play a central role in traditional food systems due to their nutritional value, extended shelf-life, organoleptic properties, and potential health benefits [3-5]. In many low- and middle-income settings, fermentation is typically carried out at household or small-scale levels using traditional methods that rely on naturally occurring indigenous microorganisms rather than commercial starter cultures for acidification and preservation [5-8].

In Uganda, consumption of fermented dairy continues to increase in both rural and urban settings [2,9]. Commonly consumed products include yogurt, locally processed cheese, homemade butter, and eshabwe, a traditional butter-based sauce widely prepared in southwestern Uganda [10,11]. These products reflect strong cultural heritage and local knowledge systems. However, their largely informal production environments often lack consistent adherence to Good Manufacturing Practices (GMP) and Good Hygiene Practices (GHP), raising concerns about microbiological safety.

Unlike industrial dairy systems, small-scale producers may lack access to pasteurization, standardized starter cultures, potable water, and routine microbial quality monitoring. Such conditions increase the likelihood of contamination by foodborne pathogens, including *Escherichia coli*, *Salmonella spp*., and *Staphylococcus aureus*, as well as other opportunistic bacteria introduced during processing and handling [10,12,13].

Culture-based methods remain the cornerstone of food safety surveillance for specific bacterial pathogens in dairy products. However, culture techniques may not detect all bacteria present within complex food matrices, particularly when organisms are present in low numbers or are not targeted by selective media. Molecular approaches, such as partial 16S rRNA gene sequencing, can provide additional taxonomic identification of predominant bacterial taxa in selected samples and complement conventional microbiological testing when interpreted appropriately [14,15].

Despite the widespread consumption of fermented milk products in southwestern Uganda, limited contemporary data exist regarding their microbiological safety in rapidly urbanizing settings such as Mbarara City. Furthermore, a few regional studies have combined culture-based pathogen detection with molecular taxonomic identification to contextualize fermentative bacteria alongside potential contaminants within the same study [10,16].

This study aimed to evaluate the microbiological safety of fermented milk products sold in Mbarara City, Uganda. The primary objective was to detect selected foodborne pathogens using culture-based methods, while the secondary objective was to identify predominant bacterial taxa using partial 16S rRNA gene sequencing. By integrating phenotypic and molecular approaches within defined methodological limits, the study provides evidence to inform practical, context-sensitive food safety interventions within traditional dairy value chains.

## Materials and methods

This cross-sectional study was conducted in Mbarara City, Uganda, between October and November 2024. Samples were collected based on product availability during the study period and represented two production contexts: commercially distributed dairy products obtained from major supermarkets and artisanal dairy products obtained from vendors operating within the Central Market. The sampling strategy was designed to capture products available to consumers across the principal retail outlets in the city and followed approaches used in comparable regional dairy safety assessments.

 A total of 192 fermented milk product samples were collected from three major supermarkets in Mbarara Town, and from vendors operating within the Central Market located in the same town. Yogurt and cheese were collected from supermarkets, whereas butter sauce (eshabwe) and locally produced butter were obtained exclusively from retail outlets within the Central Market.

Of the 192 samples collected, 175 were analyzed using culture-based microbiological methods. These included yogurt (*n *= 70), cheese (*n* = 35), butter (*n* = 35), and butter sauce (eshabwe) (*n* = 35). The remaining 17 samples, cheese (*n* = 7), yoghurt (*n *= 4), butter (*n* = 3), butter sauce (*n* = 3), were purposively selected to represent all product categories and production contexts. These samples were subjected to partial 16S rRNA gene sequencing to allow molecular identification of predominant bacterial taxa.

Samples were aseptically collected into sterile containers, labeled, and transported in cooling boxes (4 °C) to the Bacteriology Laboratory at Mbarara University of Science and Technology, Mbarara, Uganda, for microbiological and molecular analyses. All laboratory procedures were performed using standardized protocols to ensure consistency.

Microbiological analysis

High-Fat Products

For high-fat samples (e.g., cheese, butter, and eshabwe), 25 g of each sample was weighed into sterile stomacher bags, diluted in 225 mL of sterile peptone water, and homogenized at 230 rpm for two minutes using a stomacher. Serial 10-fold dilutions were prepared up to 10⁻⁷.

Detection of Microorganisms

*E. coli* was detected using ISO 7251:2005 with selective enrichment in lauryl sulphate broth and EC broth; it was confirmed using indole (Kovacs’) reagent [17]. *S. aureus* was detected using ISO 6888 using Baird-Parker Agar and the coagulase test for confirmation [18]. *Salmonella *spp. were identified using ISO 6579, which involved buffered peptone water for pre-enrichment and XLD agar for identification. Confirmation was achieved through triple sugar iron (TSI) agar, citrate, and urease biochemical tests [19]. 

DNA extraction and partial 16S rRNA gene sequencing

Sample Selection for Molecular Analysis

Seventeen representative fermented milk product samples (cheese, *n* = 7; yogurt,* n *= 4; butter, *n* = 3; eshabwe, *n* = 3) were selected for molecular analysis. The subset was selected to provide representative products for molecular identification of predominant bacterial taxa while complementing culture-based pathogen detection.

Genomic DNA Extraction

Total genomic DNA was extracted directly from homogenized food samples using the Neogen® Food DNA Isolation Kit (Neogen Corporation, Lansing, Michigan, United States) according to the manufacturer’s instructions.

DNA purity and concentration were assessed using a NanoDrop spectrophotometer (Thermo Fisher Scientific Inc., Waltham, Massachusetts, United States). Absorbance ratios (A260/A280) between 1.8 and 2.0 were considered acceptable. DNA integrity was verified by electrophoresis on 0.5% agarose gel at 100 V for 40 minutes and visualized under ultraviolet illumination.

Polymerase Chain Reaction (PCR) Amplification of 16S rRNA Gene

A partial region of the bacterial 16S rRNA gene was amplified using universal primers: (forward primer 5ʹ-CGCAAGGYTRAAACTCAA-3ʹ and reverse primer 5ʹ-ATYTCACRACACGAGCTG-3ʹ) [20]. PCR reactions were prepared in a final volume of 25 µL, which contained 12.5 μL 2x Quick load Taq master mix (New England Biolabs, Inc., Ipswich, Massachusetts, United States), 1 μL for both forward and reverse primers, 5μL template DNA, and 5.5μL nuclease free water.

Thermal cycling was performed using a MultiGene™ OptiMax Thermal Cycler (Labnet International, Edison, New Jersey, United States) under the following conditions: 95 °C for 10 minutes; 30 cycles of 94 °C for 30 seconds, 60 °C for 30 seconds, and 72 °C for 30 seconds; final extension at 72 °C for five minutes. Amplicons were visualized on a 1.5% agarose gel electrophoresis at 200 V for 45 minutes. Negative controls (no-template controls) were included in each PCR run to monitor for contamination.

Sanger Sequencing

PCR amplicons were purified using ExoSAP-IT™ PCR Product Cleanup Reagent (Thermo Fisher Scientific, Inc.) to remove residual primers and nucleotides. Purified products were sequenced using the BigDye XTerminator™ Purification Kit on a SeqStudio™ Genetic Analyzer (Thermo Fisher Scientific, Inc.), following the manufacturer’s protocol.

Sequence processing and taxonomic identification

Raw chromatograms were inspected and processed using Sequencing Analysis Software v7.0 (Thermo Fisher Scientific, Inc.). Low-quality regions and ambiguous bases were trimmed prior to analysis, and only sequences with Phred quality scores ≥ 20 were retained for downstream analysis.

High-quality sequences were compared against the National Center for Biotechnology Information (NCBI) GenBank 16S rRNA gene database using the Basic Local Alignment Search Tool (BLAST). Taxonomic assignment was based on percentage identity, query coverage, and alignment score. A minimum identity threshold of 97% was used for genus-level identification, while species-level identification was assigned only where sequence similarity supported confident resolution.

For each sample, the closest taxonomic matches were recorded, and identification was reported at the highest reliable taxonomic level supported by sequence similarity.

## Results

Culture-based microbial analysis

The fermented milk products analyzed in this study represented two production contexts: commercially distributed products (cheese and yogurt) and artisanal products (eshabwe and butter). Out of 175 fermented dairy product samples tested using conventional microbiological methods, 21 samples (12.0%) were positive for at least one of the targeted pathogens (*E. coli, S. aureus*, or *Salmonella *spp.).

Contamination by Product Type

Cheese showed the highest contamination rate, with 42.9% (15/35) of samples testing positive. In contrast, artisanal products showed lower contamination levels, with eshabwe recording 8.6% (3/35) positivity and butter showing no detectable contamination (0/35). Yogurt had the lowest contamination among the commercially distributed products, with 4.3% (3/70) of samples testing positive.

Pathogen-Specific Distribution

*E. coli* was detected in yogurt (2/70) and cheese (3/35). *S. aureus* was found in cheese (6/35) and eshabwe (3/35). *Salmonella *spp. were identified in yogurt (1/70) and cheese (6/35). The prevalence of these pathogens is noted in Table 1.

**Table 1:** Pathogen prevalence across fermented dairy products (N=175)

Product Type	Escherichia coli	Staphylococcus aureus	*Salmonella* spp.	Total Positive Samples	Sample Size	Prevalence (%)
Yogurt	2	0	1	3	70	4.30%
Cheese	3	6	6	15	35	42.90%
Butter	0	0	0	0	35	0.00%
Butter sauce	0	3	0	3	35	8.60%
Total	5	9	7	21	175	12.00%

Molecular identification of bacterial taxa

Partial 16S rRNA gene sequencing was performed on 17 representative fermented milk product samples. Sequence analysis identified a range of bacterial taxa across product categories, including lactic acid bacteria (LAB), acetic acid bacteria (AAB), and several opportunistic organisms. The taxa detected for each product category are summarized in Table 2.

**Table 2:** Dominant microbial groups by product type Species-level identification was assigned only when sequence similarity and alignment scores supported confident taxonomic resolution. In other cases, taxa are reported at the genus level. LAB: lactic acid bacteria; AAB: acetic acid bacteria

Product Type	Dominant LAB	Dominant AAB	Potential Opportunistic / Pathogenic Bacteria
Cheese	*Lactococcus* spp., *Streptococcus* spp.	–	*Streptococcus *spp.
Yogurt	*Streptococcus* spp.	*Acetobacter* spp.	*Yersinia* spp*., Klebsiella *spp.*, Leclercia *spp.,* Escherichia *spp*., Serratia *spp*., Shewanella *spp.
Butter	*Lactobacillus* spp.	*Acetobacter *spp.	*Enterobacter *spp*., Klebsiella *spp*., Escherichia *spp*., Enterococcus *spp*., Serratia *spp*.*
Eshabwe	*Lactobacillus* spp.	–	Vibrio metschnikovii

Commercially distributed dairy products (cheese and yogurt) showed diverse bacterial profiles. Cheese samples were dominated by lactic acid bacteria, with sequences corresponding primarily to *Lactococcus* spp. and *Streptococcus* spp. detected across the analyzed samples. Yogurt samples contained sequences corresponding to *Streptococcus* spp. and *Acetobacter* spp., as well as several gram-negative genera including *Yersinia* spp., *Klebsiella* spp., *Leclercia* spp., *Escherichia* spp., *Serratia* spp., and *Shewanella* spp. Some sequences also corresponded to uncultured or environmental bacterial taxa.

Artisanal dairy products (eshabwe and butter) also showed variability in the bacterial taxa detected. Eshabwe samples were dominated by LAB, with sequences corresponding to *Lactobacillus* spp., including *Lactobacillus helveticus.* In addition, sequences corresponding to *Vibrio metschnikovii* were detected in some eshabwe samples. Butter samples contained a broader range of bacterial taxa, including *Enterobacter* spp., *Serratia* spp., *Klebsiella* spp., *Escherichia* spp., *Enterococcus* spp., and acetic acid bacteria such as *Acetobacter* spp., including *Acetobacter pomorum*.

## Discussion

This study provides updated evidence on the microbiological safety of fermented milk products sold in Mbarara City, Uganda. Culture-based testing identified contamination in 12.0% of samples, with cheese exhibiting a disproportionately high prevalence (42.9%) compared to yogurt, eshabwe, and locally produced butter. This pattern suggests that contamination risk may be product-specific and linked to processing complexity rather than inherent fermentation failure.

Partial 16S rRNA gene sequencing provided complementary taxonomic identification of predominant bacterial taxa. Across product categories, sequences are most frequently aligned with lactic acid bacteria. These organisms are central to acidification, flavour development, and preservation in fermented dairy products [3,21], and their detection supports the functional integrity of traditional fermentation processes.

The concurrent identification of potential opportunistic taxa such as *Klebsiella* spp, *Enterobacter* spp, *Enterococcus* spp, and *V. metschnikovii* suggests that contamination is likely associated with post-fermentation handling, water use, or ingredient addition rather than the fermentation process itself [22,23]. This distinction is critical for intervention design, as it indicates that improving hygiene practices may be more impactful than altering traditional fermentation methods.

The findings from the eshabwe samples are particularly illustrative of potential environmental exposure pathways. This butter-based sauce is often prepared under ambient conditions using untreated water and unrefined rock salt sourced from natural saline environments, both of which may introduce environmental microorganisms [16]. Although sequences corresponding to *V. metschnikovii* were detected, species-level misclassification cannot be excluded due to the limited discriminatory resolution of partial 16S rRNA gene sequencing in mixed microbial communities. Confirmatory culture or higher-resolution genomic approaches would be required to establish the clinical relevance of this finding. Nevertheless, these observations highlight the importance of ingredient quality and water safety in non-heat-treated traditional foods [13,24,25].

Similar studies in East and West Africa have reported microbial contamination in locally processed dairy products, particularly fermented milks produced under informal conditions [26-28]. In this context, the elevated contamination in cheese samples in the present study underscores the need for targeted food safety interventions in small-scale dairy production [29]. The affected samples came from local factories that often lack standardized equipment, pasteurization, and routine microbial testing [9,30]. Contamination may occur during curd handling, water use, or packaging. All of these are critical stages where maintaining consistent hygiene is particularly challenging [8,9]. These findings highlight the need for targeted support to semi-formal producers, including food safety training and affordable interventions tailored to their operating conditions.

More broadly, these findings align with evidence from African food safety assessments. These studies demonstrate that traditional fermentation systems are not inherently unsafe; rather, risk emerges where hygiene, water quality, and standardized starter practices are inconsistent [6,12]. Importantly, the consistent presence of LAB across products indicated that fermentation remains functionally effective, even where contamination risk persisted.

The integration of culture-based detection with molecular taxonomic identification provided a broader understanding of potential microbial exposure than either approach alone. The inclusion of multiple product types across distinct production contexts (commercial and artisanal) enhances the relevance of the findings to real-world dairy value chains. In addition, the use of standardized microbiological methods strengthens the reliability and comparability of the results.

Culture-based methods confirmed the presence of viable foodborne pathogens, while sequencing identified additional taxa that may reflect environmental introduction or low-level contamination. However, because different sample subsets were used for culture and molecular analyses, the findings from the two approaches should be considered complementary rather than directly comparable.

From a public health perspective, the high contamination observed in the cheese samples underscores the need for targeted interventions within small-scale dairy value chains. Practical measures such as improving water access, hygiene training focused on curd handling and packaging, and promoting safe starter cultures may substantially reduce the risk of contamination without disrupting culturally embedded production systems. Industrial-scale regulatory standards may be unrealistic for informal producers; therefore, incremental and context-sensitive improvements are more likely to be sustainable.

As urban demand for fermented dairy products increases, strengthening microbiological surveillance and hygiene capacity within local dairy networks will be essential to prevent foodborne illness and maintain consumer confidence.

Limitations of the study and future recommendations

Several limitations should be considered when interpreting the current findings. The sampling strategy was based on product availability and may not fully represent all production systems within the study area, introducing potential selection bias. In addition, the relatively small number of samples subjected to molecular analysis limits the generalizability of the sequencing findings. Molecular analysis was conducted using Sanger sequencing of amplified 16S rRNA gene fragments, which allows identification of predominant taxa but does not provide quantitative community structure or comprehensive microbiome characterization. Furthermore, culture-based and molecular analyses were performed on different sample subsets, limiting direct comparison between methods. Culture-based testing also targeted only selected bacterial pathogens, and quantitative microbial loads (CFU/g) were not determined. Future studies incorporating broader pathogen panels, quantitative microbial enumeration, and next-generation sequencing approaches would provide a more comprehensive characterization of microbial risks in traditional dairy systems.

## Conclusions

Fermented milk products remain essential to diets and livelihoods in southwestern Uganda. This study demonstrates that while fermentation-associated lactic acid bacteria are consistently present, contamination with foodborne pathogens occurs in a subset of products, particularly those produced under informal conditions. The combined use of culture-based testing and partial 16S rRNA gene sequencing indicates that contamination is likely associated with post-fermentation handling and environmental exposure rather than failure of the fermentation process itself.

Strengthening hygiene during curd processing, improving water quality, and promoting the use of safe starter cultures represent practical and achievable strategies to reduce contamination risks without disrupting traditional production systems. Targeted, context-sensitive interventions within small-scale dairy value chains will be critical to improving food safety as demand for fermented dairy products continues to grow. Implementing these measures offers an opportunity to enhance public health outcomes while preserving culturally important food practices.

## References

[1] Bleasdale M, Richter KK, Janzen A (2021). Ancient proteins provide evidence of dairy consumption in eastern Africa. Nat Commun.

[2] Erdaw MM (2023). Contribution, prospects and trends of livestock production in sub-Saharan Africa: a review. Int J Agri Sustain.

[3] Sharma H, Ozogul F, Bartkiene E, Rocha JM (2023). Impact of lactic acid bacteria and their metabolites on the techno-functional properties and health benefits of fermented dairy products. Crit Rev Food Sci Nutr.

[4] Saleem GN, Gu R, Qu H, Bahar Khaskheli G, Rashid Rajput I, Qasim M, Chen X (2024). Therapeutic potential of popular fermented dairy products and its benefits on human health. Front Nutr.

[5] Kaur H, Kaur G, Ali SA (2022). Dairy-based probiotic-fermented functional foods: an update on their health-promoting properties. Fermentation.

[6] Leone C, Thippareddi H, Ndiaye C, Niang I, Diallo Y, Singh M (2022). Safety and quality of milk and milk products in Senegal-a review. Foods.

[7] Illikoud N, Mantel M, Rolli-Derkinderen M, Gagnaire V, Jan G (2022). Dairy starters and fermented dairy products modulate gut mucosal immunity. Immunol Lett.

[8] Rukwishuro T, Musengi A, Ndemera M, Mugiyo H, Gonde L (2025). A systematic review on indigenous starter cultures that have been applied in traditional fermentation of dairy products in sub Saharan Africa. Food Res Int.

[9] Mourad L, Safia T, Ahlam A (2022). Characterization of the consumption of milk and dairy products in the urban and rural areas of Laghouat, Algeria. Plant Arch.

[10] Ogwaro BA, Gibson H, Hill DJ (2023). Microbiological quality of typical traditional fermented Milk from northern Uganda and Western Kenya. Dairy.

[11] Waiswa D, Günlü A, Mat B (2021). Development opportunities for livestock and dairy cattle production in Uganda: a review. Res J Agri Forestry Sci.

[12] Mpatswenumugabo JP, Mukasafari MA, Ndahetuye JB, Wredle E, Båge R (2023). A systematic literature review of milk consumption and associated bacterial zoonoses in East Africa. J Appl Microbiol.

[13] El-Sayed AS, Ibrahim H, Farag MA (2022). Detection of potential microbial contaminants and their toxins in fermented dairy products: a comprehensive review. Food Analyt Method.

[14] Delikanli-Kiyak B, Yilmaz I, Guldas M (2025). Can metagenomic analyses be used effectively in safe food production?. Food Sci Nutr.

[15] You L, Yang C, Jin H (2022). Metagenomic features of traditional fermented milk products. Lwt.

[16] Xiao Z, Li X, Xue M (2022). Vibrio metschnikovii, a potential pathogen in freshwater-cultured hybrid sturgeon. Animals (Basel).

[17] Souad R, Mossadak HT, Leila B (2021). Assessing hygiene indicators in two dairies in Algeria in producing pasteurized milk. Vet World.

[18] Putri F, Surati S, Sitorus AA, Nagur KS, Cahyaningsih E, Sihotang MA, Wilasti Y (2024). Performance characteristics of the quantitative method for Staphylococcus aureus in food products corresponds to ISO 16140-3: 2021. Erudition Indonesia J Food Drug Saf.

[19] Yu W, An L, Pei XY (2021). Comparative study on the detection methods of Salmonella in infant formula milk powder and fermented milk. J Food Saf Qual.

[20] Zhang JJ, Tian JJ, Wei SS (2015). An internal reference control duplex real-time polymerase chain reaction assay for detecting bacterial contamination in blood products. PLoS One.

[21] Yassunaka Hata NN, Surek M, Sartori D, Vassoler Serrato R, Aparecida Spinosa W (2023). Role of acetic acid bacteria in food and beverages. Food Technol Biotechnol.

[22] Taale E, Ouadja B, Kadanga AK, Souha T, Amouzou KE, Tchabi A (July 2025). Microbiological quality and food poisoning risks of artisanal dairy products from the cantons of Pya and Yadè, Togo. Africa J Food Sci.

[23] Olaniran OB, Iroko OT, Famojuro TI (2022). Microbiological quality assessment of dairy product: detection of extended spectrum beta lactamase genes in bacterial isolates from yoghurt sold in Sagamu Metropolis, Ogun State, Nigeria. Dutse J Pure App Sci.

[24] Mariam SH (2021). A sampling survey of enterococci within pasteurized, fermented dairy products and their virulence and antibiotic resistance properties. PLoS One.

[25] Akinyemi MO, Ayeni KI, Ogunremi OR, Adeleke RA, Oguntoyinbo FA, Warth B, Ezekiel CN (2021). A review of microbes and chemical contaminants in dairy products in sub-Saharan Africa. Compr Rev Food Sci Food Saf.

[26] Sessou P, Keisam S, Gagara M, Komagbe G, Farougou S, Mahillon J, Jeyaram K (2023). Comparative analyses of the bacterial communities present in the spontaneously fermented milk products of Northeast India and West Africa. Front Microbiol.

[27] Bacigale SB, Ayagirwe RB, Mutwedu VB (2023). Assessing milk products quality, safety, and influencing factors along the dairy value chain in eastern Democratic Republic of the Congo. Front Sustain Food Sys.

[28] Frew M, Wolkaro T, Galmessa U (2025). Occurrence and antimicrobial susceptibility profiles of Escherichia coli and Salmonella spp. from unbranded and branded yogurt in Addis Ababa, Ethiopia. Discover Food.

[29] Hawaz H, Bottari B, Scazzina F, Carini E (2025). Eastern African traditional fermented foods and beverages: advancements, challenges, and perspectives on food technology, nutrition, and safety. Compr Rev Food Sci Food Saf.

[30] Boko C, Adje Y, Atindogbe G (2016). Evaluation of the production technologies and the microbial and physico-chemical qualities of curdled milk produced in Benin. J App Biosci.

